# We Are Dying to Eat You: Cannibalism and Disease Transmission Under Global Climate Change

**DOI:** 10.1002/ece3.74403

**Published:** 2026-09-28

**Authors:** Kale Rougeau, Bret D. Elderd

**Affiliations:** ^1^ Department of Biological Sciences Louisiana State University Baton Rouge Louisiana USA

**Keywords:** baculovirus, climate change, disease ecology, fall armyworm, resource quality

## Abstract

In terrestrial ecosystems, climate change may alter host‐pathogen dynamics along with interactions between hosts due to rising global temperatures and carbon dioxide levels. As carbon dioxide levels increase, plants may decrease their nitrogen‐carbon ratio, which may decrease plant quality for herbivores and alter species interactions. Here, we examined disease transmission, a host‐pathogen interaction, and cannibalism, an interaction between hosts, using the fall armyworm ( *Spodoptera frugiperda* ), a cannibalistic agricultural pest, and its species‐specific lethal baculovirus, *Spodoptera frugiperda* multiple nucleopolyhedrovirus (SfMNPV), under various potential challenges of climate change. We used three temperature treatments (cooler, optimal, warmer) based on the fall armyworm's thermal performance curve to simulate a changing climate and four artificial diets with various protein‐to‐carbohydrate ratios (low, equal, high, standard) to manipulate resource quality due to changes in nitrogen‐carbon ratio from increasing carbon dioxide levels. After molting to the fourth instar, experimental larvae were presented with a virus‐infected or uninfected conspecific. Cannibalism incidence and final infection status of the experimental larvae were recorded. For the experimental larvae that survived and did not become infected, pupal mass was measured as a fitness proxy. We found that higher temperatures increased cannibalism in larvae on all resource types. Larvae cannibalized more when provided with a lower protein diet. Warmer temperatures led to slightly higher disease transmission, though diet type did not influence infection results in cannibals. Changes in global temperatures and carbon dioxide levels could cause more frequent cannibalism in the fall armyworm and similar species, though disease transmission from cannibalism alone may not increase substantially. We also found significant differences in fitness estimates between each temperature and diet treatment; individuals fed more carbohydrates (lower protein) and kept at cooler temperatures had larger pupal masses, indicating potentially higher fitness, while individuals fed more protein and kept at warmer temperatures had lower fitness potential. Under climate change, increases in temperature and carbon dioxide levels affect both host‐pathogen dynamics and intraspecific interactions between hosts with varied effects on host fitness. By examining the multiple potential effects of climate change instead of examining each factor in isolation, a clearer picture of how climate change will affect ecological systems can be more thoroughly developed.

## Graphical abstract

Fall armyworm ( *Spodoptera frugiperda* ), a devastating agricultural pest that can be infected by a lethal virus, readily cannibalize conspecifics. The armyworm in the photo has just consumed a smaller conspecific—note the head capsule of the victim in its mandibles. Cannibalistic consumption can impact disease transmission. We sought to answer, how increases in temperature and decreases in resource quality tied to global climate change will affect cannibalism rates and disease transmission. As temperatures rise, cannibalism rates increase across all diet types – high protein to carbohydrate diet (A), equal protein diet (B), low protein diet (C), and standard commercial diet (D). At low temperatures, uninfected individuals were more readily cannibalized as compared to infected individuals under a low protein diet (C). Regardless of diet type, disease transmission increased as temperatures increased. Thus, as temperatures rise, cannibalism rates increase along with disease transmission. This will impact population pest outbreak dynamics as the world warms.

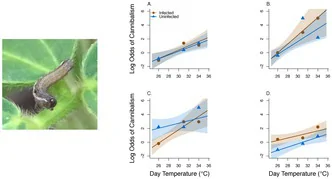

## 1 Introduction

As temperatures continue to rise worldwide due to climate change, organisms will experience changes in their behavior and physiology, which can influence species interactions (Lord et al. 2017; Rudolf and Singh 2013). An interaction of particular interest involves the transmission of diseases between hosts and their pathogens. In some host‐pathogen systems, higher temperature tends to increase disease transmission and outbreak intensity, such as with the fall armyworm ( *Spodoptera frugiperda* ) and its species‐specific baculovirus (Elderd and Reilly 2014). In other species, such as the gregarious western tent caterpillar ( *Malacosoma californicum* ), a hotter environment can reduce the spread of disease (MacDonald et al. 2023).

Beyond temperature, climate change also entails higher levels of atmospheric carbon dioxide, potentially causing nutrient dilution in resources for insect herbivores (Welti et al. 2020). As carbon content increases in plant material without a concomitant increase in nitrogen, the nitrogen‐to‐carbon ratio decreases, ultimately diluting the amount of protein consumed compared to carbohydrates (Shikano and Cory 2015). When such nutrient dilution occurs, insects need to consume more resource biomass to compensate and may be differentially affected by diseases due to the lack of important nutrients (Welti et al. 2020; Shikano and Cory 2015).

Rather than examining a single factor associated with climate change at a time (e.g., just temperature), there is a need to further examine multiple climate change stressors at once, such as both temperature and resource quality. To understand the impact of multiple stressors, a logical place to start would be systems where species are tightly linked, such as in host‐pathogen systems. This would expand our current knowledge of the impact of climate change on species interactions as well as disease dynamics.

Several lepidopteran species, such as the fall armyworm, have a tightly linked interaction with a species‐specific lethal baculoviruses that drive epizootic outbreak cycles (Cory and Myers 2003). When a fall armyworm larva ingests plant material contaminated with *Spodoptera frugiperda* multiple nucleopolyhedrovirus (SfMNPV) occlusion bodies (OBs), a species‐specific virus, the virus proliferates within the larval body until liquefaction occurs (Cory and Myers 2003). The OB‐dense cadaver fluid then contaminates the foliage that the larva is feeding on and continues the infection cycle. SfMNPV can also spread between fall armyworms when uninfected larvae cannibalize infected larvae (Van Allen et al. 2023).

In baculovirus‐driven systems, for standard horizontal disease transmission to occur, the virus needs to be consumed by a susceptible host. Warmer temperatures generally increase the feeding rate of insects, resulting in a greater chance of encountering entomopathogenic particles as the host consumes resources (Elderd and Reilly 2014), including plant material and living or dead conspecifics. However, it is not just temperature and resource quality that dictate consumption and potential infection. The infection status of a conspecific may also play a role in a fall armyworm's propensity for cannibalism (Chapman et al. 1999a). Thus, climate change could lead to changes in cannibalistic tendencies, potentially impacting both disease transmission and cannibal fitness (Chapman et al. 1999b; Ren et al. 2020).

To examine the impacts of climate change on cannibalism and disease transmission, we conducted laboratory experiments to measure cannibalism rates of infected and uninfected individual larvae and the consequential individual‐scale disease transmission of SfMNPV in fall armyworms. To simulate climate change stressors, we conducted these experiments under various temperatures and resource quality treatments. We predicted that both cannibalism and disease transmission would increase as temperature increases and protein availability decreases.

## 2 Materials and Methods

### 2.1 Study System

The fall armyworm is a multivoltine, polyphagous noctuid moth native to North and South America (Early et al. 2018). Since 2016, this highly destructive agricultural pest has invaded numerous countries throughout Africa, Asia, and Oceania (Day et al. 2017; Early et al. 2018). The larvae consume a wide variety of vegetation, posing a major threat to economically significant crops such as corn, sorghum, and sugarcane (Day et al. 2017; Early et al. 2018). The fall armyworm is considered to be a seasonal pest in the United States, with its peak outbreak intensity usually occurring during the late summer to early fall, earning its common name (Niassy et al. 2021; Philip 1928). This species does not undergo diapause, and its growth and development rates are highly influenced by temperature (Yan et al. 2022). Long‐term dynamics of this species include boom‐and‐bust cycles driven by its naturally occurring lethal pathogen, SfMNPV. SfMNPV OBs consist of a protein coat that contains multiple copies of the double‐stranded DNA virus. The OBs are large enough that they can be seen and quantified under a light microscope using a hemocytometer (Elderd 2013). Given that it is species‐specific, SfMNPV can be used as a biopesticide to manage this pest (Elderd and Reilly 2014).

Fall armyworms are highly cannibalistic during the larval stage, especially in their later instars (Chapman et al. 1999a; Mbuji et al. 2022; Sokame et al. 2023; Valicente et al. 2013). When larvae cannibalize conspecifics infected with SfMNPV, they risk becoming infected as well (Valicente et al. 2013). Cannibalism of conspecifics can have important consequences for disease outbreaks in this species (Van Allen et al. 2023) and other systems (Van Allen et al. 2017).

Together, the fall armyworm and its SfMNPV serve as an easily manipulated host‐pathogen system with economic and agricultural relevance. We used this system to investigate the potential impacts of climate change stressors on cannibalistic behavior and disease transmission.

### 2.2 Resource Quality Manipulation

We varied the ratio of protein to carbohydrates in the insect diet to mimic altered carbon content in resources under climate change (Shikano and Cory 2014, 2015). There were four total artificial diet treatments used in the experiment: high protein, equal protein/carbohydrate, low protein, and standard. The standard diet is a preformulated mix sourced from Southland Products Inc. (Lake Village, AR). It was used for all initial rearing and one diet treatment as a baseline comparison. The other three diets are macronutrient‐manipulated formulas. They were developed by modifying a recipe from Dr. Ikkei Shikano (*personal communication*), which was used in feeding experiments on the cabbage looper ( *Trichoplusia ni* ) with similar interests in examining impacts of changing resource quality (Shikano and Cory 2014, 2015). While their ratios were based on the cabbage looper's preferred host plant (Brassicaceae species), the recipe was determined to be sufficient for experimentation in the fall armyworm as well, based on ingredient comparisons. Soybeans, which are readily eaten by the fall armyworm, have a dry composition of roughly 30% protein and 30% carbohydrates, totaling 60% of the dry weight (Wrigley et al. 2004). We used these percentages as the basis for our equal protein/carbohydrate diet.

Casein (protein) and sucrose (carbohydrate) composed 60% of the total dry weight in all manipulated diets, with the protein‐to‐carbohydrate ratio of each changing depending on the treatment assigned: high protein = 50% protein to 10% carbohydrates or 5:1, equal protein/carbohydrates = 30% protein to 30% carbohydrates or 1:1, low protein = 10% protein to 50% carbohydrates or 1:5. The remaining 40% of dry ingredients consisted of the following: cellulose (25%), cholesterol (1.5%), Wesson salts (5%), ascorbic acid (1%), sorbic acid (0.5%), sodium alginate (2.5%), insect vitamin mix (3.5%), and linseed oil (1%). The recipe for making the diets used in the experiments are provided in the Supporting Information. Southland brand diet, which was used as the standard diet, has a reported dry nutritional composition of 23.5% protein and 57% carbohydrates, which is a protein‐to‐carbohydrate ratio of approximately 1:2.5.

To prepare the macronutrient‐manipulated diets, we combined the dry ingredients with room temperature water (volume = total dry mass · 2.67) and blended thoroughly. Agar (mass = total dry mass · 0.0675) was dissolved completely in boiling water (volume = total dry mass · 2.33) and then poured into the same blender. This gave a dry‐to‐wet mass ratio of about 1:5. The mixture was blended on high for 2 min. The liquid diet was poured into 1‐oz plastic cups until about a quarter filled then allowed to completely cool and solidify. We stored the diet in a refrigerator at 4°C until use. This process was developed from a combination of Dr. Shikano's instructions and the methods described by Jin et al. (2020) in a study on fall armyworm diets. The standard, Southland brand diet was made according to manufacturer's instructions and stored the same way.

### 2.3 Experimental Design

To simulate altered temperatures under climate change, we used three temperature treatments: cooler (26°C day, 16°C night), optimal (31°C day, 21°C night), and warmer (34°C day, 26°C night), each with a 14‐h daylight and 10‐h nighttime photoperiod. We determined these temperatures based on a thermal performance curve analysis for the fall armyworm in a laboratory setting (Figure S1). The averages of the day and night temperatures for the cooler, optimal, and warmer treatments correlate to just above the fall armyworm's thermal minimum, the thermal optimum, and just below the thermal maximum, respectively (Figure S1).

All fall armyworm larvae were sourced from Benzon Research Inc. (Carlisle, PA, USA) as eggs and initially hatched and reared at around 28°C in a Conviron brand growth chamber (Winnipeg, Manitoba, Canada) with a 14‐h day and 10‐h night light cycle. Larvae were reared on a standard colony diet in 1‐oz cups until the third instar molt. At this stage, we transferred half of the new third‐instar larvae to their respective diet treatments until their fourth instar molt. The treatment diet feeding period was limited to one full instar due to the poor performance of larvae on certain nutrient‐lacking diets in preliminary testing (Rougeau, *unpublished data*; Shikano, *personal communication*). A similar experimental set up was used to examine *T. ni* response to changing resource quality (Shikano and Cory 2015). The other half of the larvae were used as conspecifics for the cannibalism component and were starved as third instars after being fed only standard diet. These third instars were used to test whether the propensity for cannibalism among the fourth‐instar larvae change due to differences in diet, temperature, and viral infection status of the third instar.

The cannibalism period consisted of placing fourth‐instar larvae—after having been fed their assigned diet for one instar—in a Petri dish arena with either an infected or uninfected third‐instar conspecific. In general, larger instars will readily cannibalize smaller instars (Van Allen et al. 2023). The newly molted fourth instar larvae were placed in empty individual 1‐oz cups to starve for 24 h. An initial mass was recorded for each fourth‐instar larva prior to placement in the arenas. These individuals were the focus of the experiment.

For the conspecifics, newly molted third instar larvae were placed in empty 1‐oz cups and starved for 24 h. Half of these, labeled as uninfected, were fed 3 μL of water on a very small cube of standard diet. The other half, labeled as infected, were fed 3 μL of a 1 × 10^5^ SfMNPV occlusion bodies per μL virus suspension, a lethal dose at which 95% of third‐ and fourth‐instar larvae die (Elderd, *unpublished data*). All larvae were left to consume their cubes for 24 h.

Around 30 to 60 extra uninfected and infected conspecifics were retained for verification of successful virus infection and lack of contamination in controls. Of the extra larvae that were retained for virus verification purposes, 100% of the infected larvae died from baculovirus liquefaction, whereas no uninfected conspecifics exhibited contamination. Therefore, there was no background infection in the specimens we used. Only individuals who remained in the third instar and consumed their whole dose were selected for use in the experiment. In some cases, larvae that did not eat their entire cube had to be used, and this was noted accordingly. However, based on proof of virus verification, it did not matter if the larvae finished their entire dosed diet cube, so all conspecifics were considered to be sufficiently inoculated regardless.

We used individual arenas to allow for observation of cannibalistic behavior. Each cannibalism arena consisted of a 100 mm diameter by 15 mm tall Petri dish, a 25 mm Whatman filter paper circle moistened with DI water, and a small cube of assigned treatment diet (approximately 12.7 mm L × 12.7 mm W × 6.35 mm H) with a mass between 100 and 150 mg. We secured the Petri dishes with Parafilm once both larvae were placed inside. The arenas were then placed at their respective temperatures and monitored for cannibalism over 24 h at checkpoints of 1, 2, 4, 8, 16, and 24 h. Cannibalized infected larvae were consumed 24 to 48 h post inoculation.

After the final checks, we recorded the final mass of each fourth instar (the larger larva) and placed them back onto standard diet to be reared at their respective temperatures. We monitored the larvae daily for mortality and pupation. Virus‐caused mortality was confirmed via observation of either whole‐body liquefaction or the abundant presence of SfMNPV OBs under a microscope during dissection. We recorded the sex, mass, and eclosion status for all individuals that successfully pupated without severe deformation or desiccation (such individuals only partially pupated).

This was a fully factorial experiment with all possible combinations of the three temperature treatments, four diet treatments, and two conspecific infection treatments. Each treatment combination had 20 replicates for a total of 480 samples.

### 2.4 Data Analysis

All data analyzes were performed in R version 4.4.2 (R Core Team 2024). Data were simplified into binary response variables for both cannibalism (0 = no cannibalism by 24 h, 1 = cannibalized by 24 h) and disease transmission (0 = no virus mortality, 1 = mortality from virus). Temperature was treated as a continuous numeric value based on daytime temperature (26, 31, 34), while diet type and conspecific infection status were treated as categorical factors.

We used binomial generalized linear models (GLMs) to analyze how temperature, diet type, and conspecific infection status affected the rate of cannibalism. We constructed a series of model where each predictor variable was considered on its own, the combinations of all predictor variables (both two and three‐way) were considered additively, the combinations of all predictor variables (both two and three‐way) were considered with both additive and interaction terms, and a null model with an intercept only term (see the Supporting Information for all models considered). In total, 12 models were fit to the data. We used the small‐sample‐corrected Akaike's Information Criterion (AICc) (Akaike 1973; Sugiura 1978) for model selection due to potentially low sample sizes in proportion to the number of parameters in the models. At large sample sizes, the AICc and AIC values begin to converge, so the use of AICc is generally preferred.

For analysis of the cannibalism results, we had 12 total candidate models with all possible combinations of the independent variables: temperature, diet type, and conspecific status, along with a null model. See Table S1 in the Supporting Information document for the full list of candidate models considered for cannibalism analysis. We used the R package “AICcmodavg” to generate a table of AICc values; the model with the lowest AICc value was determined to be the best candidate model.

For the disease transmission analysis, we included only the larvae from the infected conspecific treatment that cannibalized by 24 h. We had five candidate GLMs with all possible combinations of temperature and diet type as independent variables, along with a null model. See Table S3 in the Supporting Information document for the full list of candidate models tested for disease transmission. We used an AICc model selection process as described above for cannibalism analysis. One outlier was removed from the final disease transmission analysis due to a very low sample size from minimal cannibalism skewing the data, based on diagnostic assessment in R.

We also tested a set of linear models to investigate how various factors might have affected potential fitness. We considered only individuals that successfully eclosed as functional moths, and we used their pupal masses to measure potential fitness. Pupal mass is heavily correlated with fecundity in fall armyworms, making it an accurate proxy measurement for fitness (Garvey et al. 2022). We started with 16 candidate models with combinations of the following independent variables: temperature, diet type, conspecific infection status, cannibalism, and sex, plus a null model. See Table S4 in the Supporting Information document for the full list of candidate models for fitness analysis. Again, the model with the lowest AICc value was determined to be the best fit.

Standard model diagnostics were conducted on all models. For the binomial response models, we examined linearity with respect to temperature visually and by using the Box‐Tidwell test. We looked for outliers using Cook's distance and calculated the global variance inflation factor (GVIF). For the pupal mass analysis that assumed a gaussian distribution of the error, we examined the residuals to look for heteroscedasticity. We also looked for outliers using Cook's distance and for multicollinearity by calculating the GVIF. Each of the models passed the standard assumptions associated with the analysis conducted.

## 3 Results

### 3.1 Cannibalism

Of the 12 candidate models considered for cannibalism, the full model with a three‐way interaction between temperature, diet type, and conspecific infection status had the lowest AICc score (Table S1). See Table S2 for the associated 95% confidence intervals for each of the parameters associated with the best fit model. This model accounted for 67% of the cumulative AICc weight followed by the interactive temperature and diet model with about 23% of the weight (ΔAICc = 2.18). The seven models that included temperature as a variable were the top contenders that accounted for a 100% of the AIC weight, whereas those with only diet and conspecific infection status performed poorly, as did the null model (ΔAICc = 113.80).

Across all diet types, cannibalism rates steadily increased with warmer temperatures (Figure 1). Temperature had a significant positive effect on the probability of cannibalism with a slope of 0.307, *p*‐value < 0.0009, and 95% confidence interval values of 0.134 and 0.502 in the best‐fit model. For both the high protein diet and the equal protein/carbohydrate diet, there was no difference in cannibalism between infected and uninfected larvae (Figure 1A,B). The lower protein diet treatment had the highest overall odds of cannibalism. At lower temperatures, infected individuals were not cannibalized as readily as uninfected individuals (Figure 1C). As compared to the standard diet, where infected individuals were more likely to be cannibalized as compared to uninfected larvae regardless of temperature (Figure 1D).

**FIGURE 1:**
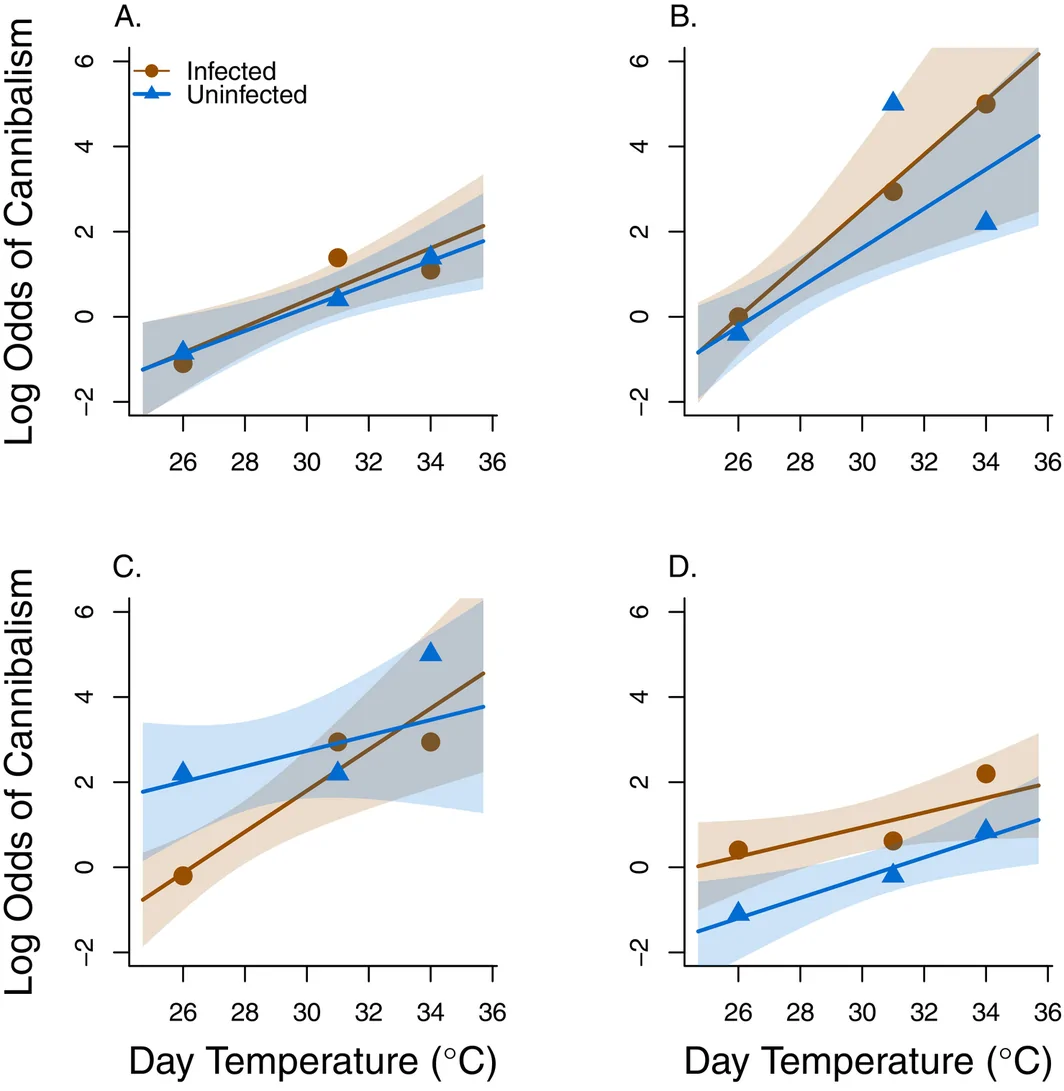
The log odds ratio of fall armyworm cannibalism on diets with different protein contents under various temperature treatments. All y‐axes represent the log odds ratios of cannibalism occurrence (log(*p*/(1 − *p*))), where *p* is the probability of cannibalism; all x‐axes represent the daytime temperature in degrees Celsius. Actual data points are shown as either circles for infected conspecific treatments or triangles for uninfected conspecific treatments. Each data point represents a treatment combination with probabilities calculated from 20 replicates. Infinite probability values (100% cannibalism) were replaced with a high proxy value of 5 for visualization purposes. The solid lines are the best fit lines derived from the regression. The 95% confidence interval bands for each diet type and infection status combination are shown as either a shaded blue fill (uninfected) or a shaded brown line fill (infected). The subfigures refer to each of the diet types (A—high protein, B—equal protein/carbohydrates, C—low protein, and D—standard diet).

### 3.2 Disease Transmission

Of the five candidate models considered for disease transmission, the temperature‐only model had the lowest AICc score (Table S3). Temperature alone accounted for 62% of the AICc weight. The null model also had reasonable support (ΔAICc = 2.40, AICc weight = 19%) along with the temperature and diet additive model (ΔAICc = 2.89, AICc weight = 15%). The remaining models had very little support.

The temperature‐only GLM had a slope coefficient of 0.117 (standard error = 0.056), and temperature had a significant effect on disease transmission as the confidence interval values did not overlap zero (*p*‐value = 0.04; 95% confidence interval values = 0.008 and 0.233). Temperature caused a slight positive increase in disease transmission (Figure 2).

**FIGURE 2:**
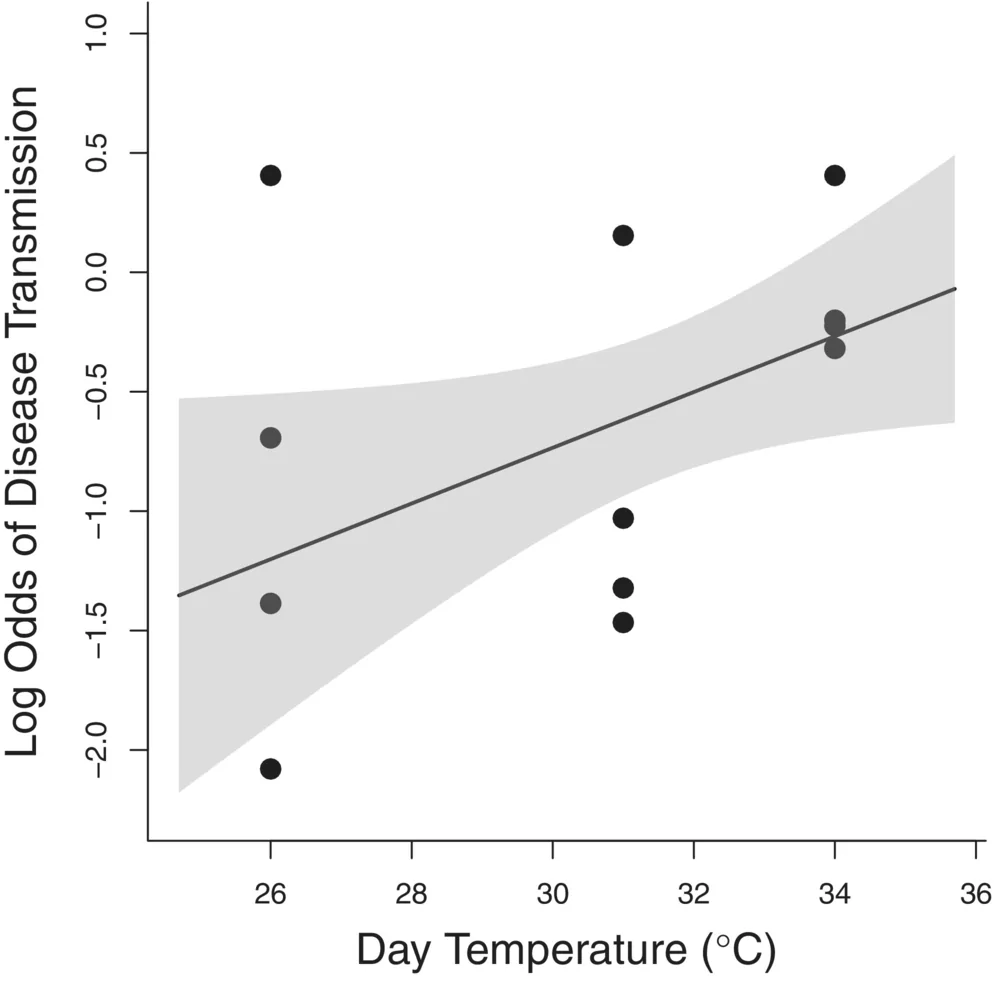
Probability of fall armyworm disease transmission over temperature for all combined diet types. Only larvae that cannibalized infected conspecifics were included for analysis of disease transmission results. The data points represent the log odds ratio (probabilities) of infection occurrence (log(*p*/(1 − *p*))) after cannibalism. Each data point represents a treatment combination with varying numbers of replicates, depending on how many individuals successfully cannibalized infected conspecifics within 24 h. The solid line represents the binomial generalized linear regression best‐fit model with daytime temperature as the predictor of final infection status. The shaded region around the line shows the 95% confidence intervals for the model.

Only two individuals across all treatments became infected without consuming their conspecific, so we excluded those two outliers from the disease transmission analysis. It is possible that those individuals consumed viral particles passed through frass or other debris, and we wanted to focus solely on virus transmission from direct cannibalism rather than trace contamination. Therefore, all infected individuals in our analysis contracted the disease through the cannibalism of an infected conspecific.

### 3.3 Fitness

Of the 16 candidate models tested for potential fitness, the model with a two‐way interaction between temperature and diet type had the lowest AICc score (Table S4). This model accounted for 85% of the cumulative AICc weight, followed by the model that allows for an interaction between temperature, diet, and conspecific infection status model with about 13% of the weight (ΔAICc = 3.83). The other 14 models accounted for the remaining 2%; cannibalism status and pupal sex did not affect pupal mass.

As temperatures increased, the pupal mass declined except for individuals on the standard diet where pupal mass did not change across temperature treatments (Figure 3). Across diet treatments, the low protein diet had a higher pupal mass (Figure 3C) especially as compared to the high protein diet (Figure 3A). A total of 199 moths were analyzed, and the average pupal mass overall was 160 ± 3 mg. Moths from the low protein diet treatment (*n* = 62) had the highest pupal masses with a mean of about 184 ± 5 mg, whereas moths from the high protein diet treatment were fewer in number (*n* = 27) and had the lowest pupal masses with a mean of about 123 ± 9 mg (Figure 3). Individuals in the warmer temperature treatment had lower pupal masses across all macronutrient‐manipulated diets, whereas there was little difference in pupal mass across temperatures for the standard diet.

**FIGURE 3:**
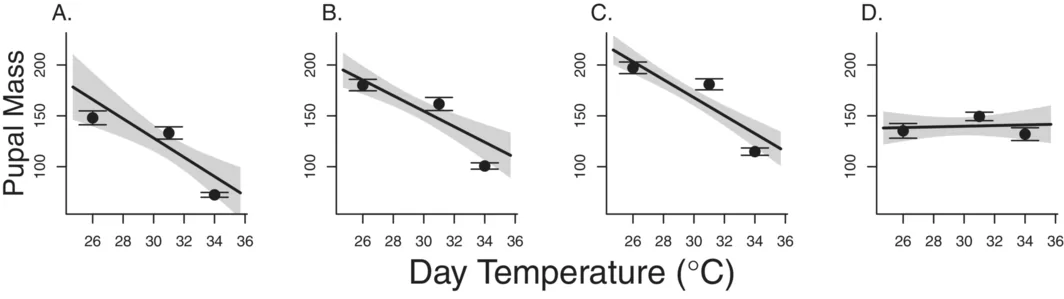
Pupal masses of successfully eclosed moths fed diets with different protein contents and kept at various temperature treatments. The data points represent the mean of a particular treatment. The error bars are the standard errors associated with the treatment. The solid lines are the best‐fit lines, and the shaded regions are the 95% Confidence Intervals. The subfigures refer to each of the diet types (A—high protein, B—equal protein/carbohydrates, C—low protein, and D—standard diet).

## 4 Discussion

### 4.1 Cannibalism

We found that the interaction between temperature, diet type, and conspecific infection status best explained the odds of being cannibalized (Table S1). Individuals kept at warmer temperatures were more likely to cannibalize (Figure 1), potentially due to higher metabolic and energetic demands. Larvae cannibalized more frequently when fed the lower protein diets (Figure 1C), indicating a need to supplement protein from their conspecifics. In general, at higher temperatures, infection status did not change the chance of being cannibalized. Interestingly, at lower temperatures for the low protein diet, infected conspecifics had a lower likelihood of being cannibalized (Figure 1C). Thus, at higher temperatures—and particularly for the low protein diet—cannibalism increased, potentially due to lower protein availability and higher energy demands. This demonstrates the importance of the interaction between temperature, diet type, and conspecific infection status on the propensity for cannibalism rather than temperature alone.

For this particularly devastating pest (Day et al. 2017; Early et al. 2018) and its species‐specific lethal virus, we expect that climate change will cause an increase in cannibalism during warmer periods due to a combined effect of energy demands and nutrient dilution as nitrogen to carbon ratios decrease because of increased atmospheric carbon dioxide. As conspecifics often harbor pathogens, increased temperatures along with declining resource quality could lead to further spread of disease in this system and perhaps others. However, the cannibalism of infected conspecifics does not always lead to infection (Van Allen et al. 2023), which would lead to the removal of pathogen from the system. Thus, an increase in cannibalism will not necessarily lead to increases in transmission and could potentially decrease disease spread (Van Allen et al. 2017).

### 4.2 Disease Transmission

In terms of pathogen transmission post‐cannibalism, temperature alone increased the probability of becoming infected (Table S3). However, in comparison to the best‐fit cannibalism model, temperature is a much lower driver of disease transmission than cannibalism. The estimated slope associated with disease transmission (Figure 2, slope = 0.117 with a 95% confidence interval of 0.0081 to 0.2328, *p* < 0.041) was almost one‐third the size of the coefficient associated with cannibalism (Figure 1, slope = 0.307 with a 95% confidence interval of 0.1343 to 0.5023, *p* < 0.001). Although, the confidence intervals overlap to a degree. Given that the infected conspecifics were cannibalized 24 to 48 h after viral inoculation, transmission would have undoubtedly increased if the conspecifics had been consumed later in the infection process as the virus load increased. Additionally, only temperature significantly affected disease transmission, unlike cannibalism, which was affected by both temperature and resource quality as potential stressors. While warming temperatures from climate change play somewhat of a role in disease transmission dynamics in this system, especially through the increase in cannibalism, it has a relatively low direct effect on infection risk and is not compounded by changes in resource quality. As suggested by Elderd and Reilly (2014), it is likely that temperature is actually decreasing the heterogeneity of disease risk across individuals rather than simply increasing the disease transmission rate itself.

It is also possible that the temperature changes are partially mitigated by the speed of molting in the fall armyworm, as far as infection risk is concerned. Higher temperatures increase fall armyworm metabolism and shorten the duration between larval instars (Elderd and Reilly 2014). A more demanding metabolism requires higher energy expenditures, reducing an organism's resources available for immune function and seemingly putting hotter individuals at a higher risk for lethal infection due to the trade‐off (Tao et al. 2023). However, each time a fall armyworm larva molts, it sheds the lining of its midgut epithelial cells, expelling recently consumed infectious particles and potentially preventing the establishment of the virus (Elderd and Reilly 2014; Engelhard et al. 1994). Although SfMNPV interrupts molting, a larva may shed its midgut too quickly at higher temperatures for the virus to take over, partially mitigating the higher risk of infection at warmer temperatures. Thus, there are confounding factors that may either raise or dampen the effects of temperature on transmission. For cannibalistic individuals, the risk of becoming infected increases as temperature rises but to a limited extent (Figure 2).

While higher temperature leads to slightly higher rates of disease transmission in this system (Figure 2), other host‐pathogen systems experience the opposite effect. For example, warmer temperatures reduce the spread of the western tent caterpillar's ( *Malacosoma californicum* ) species‐specific baculovirus, McplMNPV, as the gregarious larvae disperse more in warmer weather rather than congregating close together (MacDonald et al. 2023). In the case of the spongy moth ( *Lymantria dispar* ) and its fungal pathogen *Entomophaga maimaiga*, the warmer, drier conditions from climate change reduce infection prevalence in the species (Liu et al. 2025). In general, climate change is likely to affect host‐pathogen dynamics in different ways depending on factors such as host behavior, host physiology, and mode of disease spread.

### 4.3 Fitness

The model with an interaction between temperature and diet type best predicted the potential fitness results of the larvae (Table S4). The pupae from the low protein diet treatment had the highest pupal masses, indicating higher potential fitness, especially at the cooler temperature (Figure 3). Given that the individuals fed a lower protein diet also cannibalized at higher rates, it is possible that cannibals gained a fitness advantage from consuming their conspecifics. However, this would only apply to nutritionally disadvantaged individuals, as cannibalism status was not found to be a strong predictor of pupal mass (Table S4). Pupal mass was also lower in high temperatures for all macronutrient‐manipulated diets but not the standard formula (Figure 3), indicating combined stress of high temperature with resource quality.

Chapman et al. (1999b) found that fall armyworms experienced fitness consequences after cannibalizing during high food availability and fitness benefits after cannibalizing during low food availability. In our study, the larvae were only briefly exposed to conspecifics and resource challenges, making the potential fitness implications less drastic. However, our results further corroborate that resource quality often determines whether an individual experiences either fitness advantages or consequences from cannibalism. It is also possible that a higher carbohydrate intake directly contributes to a higher pupal mass regardless of cannibalistic behavior, especially when considering the model selection results (Table S4).

While baculoviruses frequently cause a lethal infection, sub‐lethal infections still occur. Such sub‐lethal infections do not lead to direct virus‐induced mortality but still entail a viral load being maintained within the host, potentially stressing the host's body and leading to indirect mortality, changes in behavior, reduced fitness, or even transgenerational (vertical) transmission (Williams et al. 2017). In our analysis of fitness results, we did not find differences in pupal masses based on conspecific infection status or cannibalism, indicating a lack of significant effects from potential sub‐lethal viral loads in surviving individuals.

## 5 Conclusions

With climate change continuously causing more extreme temperatures and diminished resource quality, we expect an increase in cannibalistic behavior in this experimental system, but this may also hold for other readily cannibalistic, herbivorous insect species. With higher rates of cannibalism, we also expect slightly more disease transmission from cannibalism, compounded by a slight increase in infection risk from temperature alone. In the case of carbohydrate‐rich, protein‐poor resources, some individuals could also experience a fitness advantage, while others may experience fitness consequences with higher protein diets or warmer temperatures. A natural extension of this work would be to test these ideas in a field system to see how the interaction between warmer temperatures and decreased resource quality affect population dynamics. The latter, however, is relatively hard to manipulate. Another way forward is by modeling these dynamics in silico (e.g., Van Allen et al. 2023). Regardless of which future avenues are explored, it has been predicted, based on climate change data, that fall armyworms will proliferate in certain geographic regions and potentially go locally extinct in others, particularly in areas already near their critical thermal maximum (Early et al. 2018). Changes in cannibalistic behavior and baculovirus transmission may also affect how the species spreads globally in the future. As temperatures warm, we should see the direct effects of climate change via increased rates of cannibalism as well as the indirect effects of climate change through altered resource quality and changes in disease transmission.

## Author Contributions

**Kale Rougeau:** conceptualization (lead), data curation (lead), formal analysis (equal), methodology (lead), writing – original draft (lead), writing – review and editing (lead). **Bret D. Elderd:** conceptualization (supporting), data curation (supporting), formal analysis (equal), methodology (supporting), writing – original draft (supporting), writing – review and editing (supporting).

## Funding

The work was supported by USDA (Grant 2019‐67014‐29919).

## Disclosure

Inclusion Statement: Our study was conducted by scientists from the country where the study was carried out.

## Ethics Statement

This work did not require ethical approval from an animal welfare committee.

## Conflicts of Interest

The authors declare no conflicts of interest.

## Supporting information


**Figure S1:** A thermal performance curve for the fall armyworm ( *Spodoptera frugiperda* ). “Temp” on the x‐axis represents temperature in degrees Celsius. “Survivorship” on the y‐axis represents the proportion of surviving individuals in each temperature treatment.
**Table S1:** Candidate models and AICc model selection results for cannibalism. “Temp” is short for temperature, “diet” refers to diet type, and “con” means conspecific infection status. A “·” between the model terms represents an additive and interaction between those terms. For example, temp · diet corresponds to the model in R where cannibalism ~ temperature + diet + temperature: diet. K represents the number of model parameters. The model with the lowest AICc value is the best candidate for representing the data. ΔAICc is the difference between a given model's AICc value and the best candidate. “LL” is the estimate of model log‐likelihood. The “AICc Wt.” column consists of individual weight values for each model, while “Cum. Wt.” is the cumulative weight, adding up to 1 total. Sample size for the analysis was 480 individuals.
**Table S2:** The 95% confidence interval estimates for the effect of day temperature on cannibalism for each treatment combination (diet type and conspecific infection status) using binomial GLMs. Asterisks (*) indicate statistically significant *p*‐values (*α* < 0.05) for each subset‐specific model.
**Table S3:** Candidate models and AICc model selection results for disease transmission. Only larvae that cannibalized infected conspecifics were included in this analysis, so conspecific infection status and cannibalism status were not included as variables. “Temp” is short for temperature, and “diet” refers to diet type. A “·” between the model terms represents an additive and interaction between those terms. For example, temp · diet corresponds to the model in R where cannibalism ~ temperature + diet + temperature: diet. K represents the number of model parameters. The model with the lowest AICc value is the best candidate for representing the data. ΔAICc is the difference between a given model's AICc value and the best candidate. “LL” is the estimate of model log‐likelihood. The “AICc Wt.” column consists of individual weight values for each model, while “Cum. Wt.” is the cumulative weight, adding up to 1 total. Sample size for the analysis was 175 individuals.
**Table S4:** Candidate models and AICc model selection results for potential fitness. Only successfully eclosed moths were included, and pupal mass was used as a proxy measurement for potential fitness. “Temp” is short for temperature, “diet” refers to diet type, “con” means conspecific infection status, “cann” refers to cannibalism status at 24 h, and “sex” is the sex of the moth as determined during the pupal stage. A “·” between the model terms represents an additive and interaction between those terms. For example, temp · diet corresponds to the model in R where cannibalism ~ temperature + diet + temperature: diet. K represents the number of model parameters. The model with the lowest AICc value is the best candidate for representing the data. ΔAICc is the difference between a given model's AICc value and the best candidate. “LL” is the estimate of model log‐likelihood. The “AICc Wt.” column consists of individual weight values for each model, while “Cum. Wt.” is the cumulative weight, adding up to 1 total. Sample size for the analysis was 243 individuals.

## Data Availability

The data along with the associated R code to complete the analysis and construct the figures are available in a Dryad Repository, https://doi.org/10.5061/dryad.jdfn2z3sh.
