# Supplementary material for: We Are Dying to Eat You: Cannibalism and Disease Transmission Under Global Climate Change
Source: Ecol Evol. 2026 Sep 28;16(10):e74403. doi: 10.1002/ece3.74403 (PMC13619740; doi:10.1002/ece3.74403)
Supplement: Supplementary file 1 [file ECE3-16-e74403-s001.docx]

**Supporting Information**

Figure S1. A thermal performance curve for the fall armyworm (*Spodoptera frugiperda*). “Temp” on the x-axis represents temperature in degrees Celsius. “Survivorship” on the y-axis represents the proportion of surviving individuals in each temperature treatment.

Table S1. Candidate models and AICc model selection results for cannibalism. “Temp” is short for temperature, “diet” refers to diet type, and “con” means conspecific infection status. A "·" between the model terms represents an additive and interaction between those terms. For example, temp · diet corresponds to the model in R where cannibalism ~ temperature + diet + temperature:diet. K represents the number of model parameters. The model with the lowest AICc value is the best candidate for representing the data. ΔAICc is the difference between a given model’s AICc value and the best candidate. “LL” is the estimate of model log-likelihood. The “AICc Wt.” column consists of individual weight values for each model, while “Cum. Wt.” is the cumulative weight, adding up to 1 total. Sample size for the analysis was 480 individuals.

| **Model** | **K** | **AICc** | **ΔAICc** | **LL** | **AICc Wt.** | **Cum. Wt.** |
| --- | --- | --- | --- | --- | --- | --- |
| temp · diet · con | 16 | 470.26 | 0.00 | -218.54 | 0.67 | 0.671 |
| temp · diet | 8 | 472.44 | 2.18 | -228.07 | 0.226 | 0.897 |
| temp + diet | 5 | 475.18 | 4.91 | -232.53 | 0.058 | 0.954 |
| temp + diet + con | 6 | 475.64 | 5.37 | -231.73 | 0.046 | 1.000 |
| temp | 2 | 511.10 | 40.83 | -253.54 | 9.12e-10 | 1.000 |
| temp + con | 3 | 511.68 | 41.42 | -252.82 | 6.80e-10 | 1.000 |
| temp · con | 4 | 512.16 | 41.90 | -252.04 | 5.35e-10 | 1.000 |
| diet · con | 8 | 548.09 | 77.82 | -265.89 | 8.46e-18 | 1.000 |
| diet | 4 | 554.12 | 83.86 | -273.02 | 4.14e-19 | 1.000 |
| diet + con | 5 | 554.86 | 84.59 | -272.36 | 2.87e-19 | 1.000 |
| null | 1 | 583.26 | 113.00 | -290.63 | 1.94e-25 | 1.000 |
| con | 2 | 584.07 | 113.80 | -290.02 | 1.30e-25 | 1.000 |

Table S2. The 95% confidence interval estimates for the effect of day temperature on cannibalism for each treatment combination (diet type and conspecific infection status) using binomial GLMs. Asterisks (*) indicate statistically significant p-values (α < 0.05) for each subset-specific model.

| **Diet Type** | **Infection Treatment** | **Lower Bound** | **Upper Bound** | **p-value** |
| --- | --- | --- | --- | --- |
| high protein | uninfected | 0.237 | 0.316 | 0.047* |
| high protein | infected | -0.011 | 0.605 | 0.310 |
| equal protein/carbs | uninfected | -0.645 | 1.450 | 0.589 |
| equal protein/carbs | infected | 0.571 | 0.671 | 0.026* |
| low protein | uninfected | -0.171 | 0.800 | 0.425 |
| low protein | infected | 0.090 | 0.744 | 0.242 |
| standard protein | uninfected | 0.149 | 0.325 | 0.120 |
| standard protein | infected | -0.046 | 0.457 | 0.355 |

Table S3. Candidate models and AICc model selection results for disease transmission. Only larvae that cannibalized infected conspecifics were included in this analysis, so conspecific infection status and cannibalism status were not included as variables. “Temp” is short for temperature, and “diet” refers to diet type. A "·" between the model terms represents an additive and interaction between those terms. For example, temp · diet corresponds to the model in R where cannibalism ~ temperature + diet + temperature:diet. K represents the number of model parameters. The model with the lowest AICc value is the best candidate for representing the data. ΔAICc is the difference between a given model’s AICc value and the best candidate. “LL” is the estimate of model log-likelihood. The “AICc Wt.” column consists of individual weight values for each model, while “Cum. Wt.” is the cumulative weight, adding up to 1 total. Sample size for the analysis was 175 individuals.

| **Model** | **K** | **AICc** | **ΔAICc** | **LL** | **AICc Wt.** | **Cum. Wt.** |
| --- | --- | --- | --- | --- | --- | --- |
| temp | 2 | 228.32 | 0.00 | -112.12 | 0.62 | 0.62 |
| null | 1 | 230.72 | 2.40 | -114.35 | 0.19 | 0.80 |
| temp + diet | 5 | 231.20 | 2.89 | -110.42 | 0.15 | 0.95 |
| diet | 4 | 233.78 | 5.46 | -112.77 | 0.04 | 0.99 |
| temp · diet | 8 | 236.30 | 7.98 | -109.72 | 0.01 | 1.00 |

Table S4. Candidate models and AICc model selection results for potential fitness. Only successfully eclosed moths were included, and pupal mass was used as a proxy measurement for potential fitness. “Temp” is short for temperature, “diet” refers to diet type, “con” means conspecific infection status, “cann” refers to cannibalism status at 24 hours, and “sex” is the sex of the moth as determined during the pupal stage. A "·" between the model terms represents an additive and interaction between those terms. For example, temp · diet corresponds to the model in R where cannibalism ~ temperature + diet + temperature:diet. K represents the number of model parameters. The model with the lowest AICc value is the best candidate for representing the data. ΔAICc is the difference between a given model’s AICc value and the best candidate. “LL” is the estimate of model log-likelihood. The “AICc Wt.” column consists of individual weight values for each model, while “Cum. Wt.” is the cumulative weight, adding up to 1 total. Sample size for the analysis was 243 individuals.

| **Model** | **K** | **AICc** | **ΔAICc** | **LL** | **AICc Wt.** | **Cum. Wt.** |
| --- | --- | --- | --- | --- | --- | --- |
| temp · diet | 9 | 1994.85 | 0.00 | -987.95 | 0.85 | 0.85 |
| temp · diet · con | 17 | 1998.68 | 3.83 | -980.65 | 0.13 | 0.98 |
| temp + diet + con + cann + sex | 9 | 2004.44 | 9.58 | -992.74 | 0.01 | 0.99 |
| temp + diet | 6 | 2004.47 | 9.62 | -996.02 | 0.01 | 0.99 |
| temp + diet + can | 7 | 2005.23 | 10.37 | -995.32 | 0.00 | 1.00 |
| temp + diet + can + cann | 8 | 2006.88 | 12.02 | -995.06 | 0.00 | 1.00 |
| temp · diet · con · cann | 30 | 2011.37 | 16.61 | -970.15 | 0.00 | 1.00 |
| diet + con | 6 | 2033.74 | 38.89 | -1010.65 | 0.00 | 1.00 |
| diet · con | 9 | 2036.20 | 41.35 | -1008.62 | 0.00 | 1.00 |
| diet | 5 | 2036.22 | 41.37 | -1012.95 | 0.00 | 1.00 |
| temp | 3 | 2037.75 | 42.90 | -1015.81 | 0.00 | 1.00 |
| temp · diet · con · cann · sex | 56 | 2046.18 | 51.33 | -944.61 | 0.00 | 1.00 |
| sex | 3 | 2075.97 | 81.11 | -1034.92 | 0.00 | 1.00 |
| null | 2 | 2078.55 | 83.70 | -1037.24 | 0.00 | 1.00 |
| con | 3 | 2079.04 | 84.19 | -1036.46 | 0.00 | 1.00 |
| cann | 3 | 2080.54 | 85.68 | -1037.21 | 0.00 | 1.00 |

**Experimental FAW Diet Recipe**

| **Ingredients** | **Treatment B (equal protein/carbs)**  **Single \| Double \| Triple** |
| --- | --- |
| Casein | 30 g \| 60 g \| 90 g |
| Sucrose | 30 g \| 60 g \| 90 g |
| Cellulose | 25 g \| 50 g \| 75 g |
| Cholesterol | 1.5 g \| 3 g \| 4.5 g |
| Wesson salts | 5 g \| 10 g \| 15 g |
| Ascorbic acid | 1 g \| 2 g \| 3 g |
| Sorbic acid | .50 g \| 1 g \| 1.5 g |
| Sodium alginate | 2.5 g \| 5 g \| 7.5 g |
| Vanderzant vitamin mix | 3.5 g \| 7 g \| 10.5 g |
| Linseed oil | 1 g \| 2 g \| 3 g |
| **Total Dry Diet** | **100 g \| 200 g \| 300 g** |
| Agar | 6.75 g \| 13.5 g \| 20.25 g |
| Water (room temp) | 267 mL \| 534 mL \| 801 mL (800) |
| Water (agar-containing, boiling) | 233 mL \| 466 mL \| 699 mL (700) |
| **Total Grams** | **606.75 g \| 1,213.5 g \| 1,820.25 g** |

The recipes for Treatment A and C consist of the same ingredients above (Treatment B) but with modified casein to sucrose ratios as specified in the table below for a single batch.

| **Weight (grams) per Treatment** | **A** | **B** | **C** |
| --- | --- | --- | --- |
| **Casein (protein)** | 50 | 30 | 10 |
| **Sucrose (carbs)** | 10 | 30 | 50 |

This diet recipe was modified from a base recipe provided by Dr. Shikano, as used in Shikano and Cory (2014). Linseed oil was substituted for wheat-germ oil, as this is standard in commercial fall armyworm diets, no antibiotics were added, and dry ingredients were not sterilized prior to use.

The general process used to blend the diet was derived from Jin et al. (2020). The dry ingredients besides the agar were mixed in a blender with room temperature distilled water. The agar and the boiled distilled water were added to the blender and the entire mix was blended for approximately 2 minutes. The mixture was poured into a plastic container and allowed to cool. After cooling, it was stored in a refrigerator at 4°C. When ready for use, the diet was cut into pieces and kept at room temperature for 2 hours before being used in the experiment.

Shikano, I., & Cory, J. S. (2014). Dietary Mechanism behind the Costs Associated with Resistance to	*Bacillus thuringiensis* in the Cabbage Looper, *Trichoplusia ni*. PLoS ONE, 9(8), e105864. <https://doi.org/10.1371/journal.pone.0105864>

Jin, T., Lin, Y., Chi, H., Xiang, K., Ma, G., Peng, Z., & Yi, K. (2020). Comparative Performance of the Fall Armyworm (Lepidoptera: Noctuidae) Reared on Various Cereal-Based Artificial Diets. *Journal of Economic Entomology, 113*(6), 2986–2996. <https://doi.org/10.1093/jee/toaa198>
